# Development of motion speed perception from infancy to early adulthood: a high-density EEG study of simulated forward motion through optic flow

**DOI:** 10.1007/s00221-021-06195-5

**Published:** 2021-08-21

**Authors:** Stefania Rasulo, Kenneth Vilhelmsen, F. R. (Ruud) van der Weel, Audrey L. H. van der Meer

**Affiliations:** Developmental Neuroscience Laboratory, Department of Psychology, Norwegian University of Science and Technology (NTNU), Trondheim, Norway

**Keywords:** Motion speed perception, Optic flow, High-density EEG, Visual evoked potential (VEP), Temporal spectral evolution (TSE), Lifespan visual motion perception

## Abstract

This study investigated evoked and oscillatory brain activity in response to forward visual motion at three different ecologically valid speeds, simulated through an optic flow pattern consisting of a virtual road with moving poles at either side of it. Participants were prelocomotor infants at 4–5 months, crawling infants at 9–11 months, primary school children at 6 years, adolescents at 12 years, and young adults. N2 latencies for motion decreased significantly with age from around 400 ms in prelocomotor infants to 325 ms in crawling infants, and from 300 and 275 ms in 6- and 12-year-olds, respectively, to 250 ms in adults. Infants at 4–5 months displayed the longest latencies and appeared unable to differentiate between motion speeds. In contrast, crawling infants at 9–11 months and 6-year-old children differentiated between low, medium and high speeds, with shortest latency for low speed. Adolescents and adults displayed similar short latencies for the three motion speeds, indicating that they perceived them as equally easy to detect. Time–frequency analyses indicated that with increasing age, participants showed a progression from low- to high-frequency desynchronized oscillatory brain activity in response to visual motion. The developmental differences in motion speed perception are interpreted in terms of a combination of neurobiological development and increased experience with self-produced locomotion. Our findings suggest that motion speed perception is not fully developed until adolescence, which has implications for children’s road traffic safety.

**Supplementary Information:**

The online version contains supplementary material available at 10.1007/s00221-021-06195-5.

## Introduction

Optic flow is the pattern of visual information produced by self-motion (Gibson 1979/2015). Essential to navigating in the real world, optic flow provides information about the relative movement of objects, and their speeds in relation to the observer (Vilhelmsen et al. 2019). When we move around, the environment changes according to our movements, and optic flow is crucial when controlling heading direction (Bruggeman et al. 2007; Warren et al. 2001), stabilizing posture (Bertenthal et al. 1997; Higgins et al. 1996), and estimating time-to-contact of an approaching object (Kayed and Van der Meer 2009; Vaina and Rushton 2000; Van der Meer et al. 1994; Wilkie and Wann 2003).

Optic flow information is processed by the medial superior temporal (MST) area of the dorsal visual stream (Duffy 1998; Holliday and Meese 2008; Yu et al. 2010). The processing of radial motion involves the human medial temporal/visual area 5 (hMT/V5) (Dukelow et al. 2001; Morrone et al. 2000; Smith et al. 2006), while global motion elicits activity in parietal areas and visual area V3a (Wattam-Bell 1991, 2010).

When we walk in a forward direction, the point we are facing expands, and a flow radiates from its centre. When we walk backward, on the other hand, the same point will contract and the flow will radiate toward the point (Bruce et al. 2003). If our speed increases, the velocity in the optic flow will change accordingly, leading to changes in brain activity (Vilhelmsen et al. 2015) that show a gradual developmental progression through childhood (Manning et al. 2019). Thus, we rely on optic flow every time we move (Warren et al. 2001), and it is a crucial source of information to control our walking direction and speed.

In the past few years, electroencephalography (EEG) has been the focus of several visual motion studies, due to its high temporal accuracy. During visual processing, EEG activity is dominated by N2, an ERP component related to visual motion perception. N2 is a negative-going deflection typically observed in posterior occipital and parietal areas, and responds to changes in visual motion (Luck 2014). Previous studies found N2 around 130–150 ms after stimulus onset in adults (Probst et al. 1993; Kremláček et al. 2004), and after around 180–220 ms in infants aged 8 months (Van der Meer et al. 2008).

Previous research argued that infants are already able to discriminate between approaching and receding objects shortly after birth (Gilmore et al. 2007), and that the development of their visual motion perception is closely linked to their locomotor experience, since the active exploration of the surroundings leads to an improvement of the visual system (Agyei et al. 2015; Gilmore et al. 2004; Higgins et al. 1996). During the first year of life, infants process optic flow information slower than adults, showing higher N2 latencies (Van der Meer et al. 2008), lower frequency neuronal oscillations (Agyei et al. 2016a; Stroganova et al. 1999), and an initial higher sensitivity to faster motion speeds (Gilmore et al. 2016). In addition, other studies suggested that infants’ global integration mechanisms of motion are still immature at 4–6 months (Hou et al. 2009).

Due to its role in traffic dynamics, optic flow has also been the focus of several studies on road-crossing safety. Wann et al. (2011) reported that children at 6–11 years are not yet able to properly assess the speed of an approaching vehicle, resulting in impaired ability of safely crossing a busy road. Moreover, children aged 6–12 years show differential mechanisms of motion processing when compared to adults (Manning et al. 2019). According to the World Health Organization (2013), traffic accidents are a major cause of serious injuries among children and are ranked as the second most frequent cause of death in children between 10 and 14 years worldwide. Research suggests that at the age of 12, pedestrians are at most risk of accidents (Owen and Fosdick 2013) since an adult-level response to visual motion does not occur until 16–18 years of age (Hadad et al. 2015).

The aim of this study was to investigate the development of motion speed perception in response to simulated forwards motion from infancy to early adulthood, using analyses of visual evoked potentials (VEPs) and time–frequency oscillations (TSE, temporal spectral evolution). Latency differences and frequency changes in oscillations between prelocomotor and locomotor infants, 6- and 12-year-olds, and young adults were investigated in parietal and occipital areas. A previous study (Vilhelmsen et al. 2019) used the same stimulus with driving speeds between 25 and 75 km/h, and found that prelocomotor infants at 4–5 months displayed equally long latencies compared to older infants for the high driving speeds only, while adults showed significantly shorter latencies overall. Furthermore, low-frequency-induced desynchronization in response to visual motion was found in both infant groups in the theta and alpha bands, while adults displayed consistent high-frequency EEG rhythms in the beta band. As the driving speeds used in the previous study turned out to be too high for infants, the present study used much lower speeds, representative of walking, jogging, and cycling (approximately 5, 10 and 20 km/h), to determine whether and if so when the youngest participants would be able to differentiate between more ecologically valid motion speeds. The sample has also been enlarged to include 6- and 12-year-old children, to give a better understanding of speed perception from a developmental perspective. It was, therefore, hypothesized that N2 latency for motion would decrease with age, with no differentiation between speeds in prelocomotor infants. Moreover, desynchronized oscillatory activity in response to visual motion was expected to progressively increase in frequency from the theta band (4–8 Hz) in early infancy to the beta band (12–30 Hz) in adulthood since neuronal assemblies have been shown to oscillate at higher frequency as the brain matures (Agyei et al. 2015), with the fastest oscillations found in adults (Van der Meer et al. 2008; Vilhelmsen et al. 2015).

## Materials and methods

### Participants

A total of 40 healthy participants, divided by age into five groups each consisting of eight participants, were recruited for this study. The study followed a cross-sectional design, testing infants at 4–5 months, infants at 9–11 months, children at 6 years, adolescents at 12 years, and young adults between 21 and 29 years of age.

Infants were recruited by contacting parents through birth announcements in the local newspaper. All the children at 6 years were tested as infants about 5 years before on different but related experiments, and came in for a follow-up session. 12-year-olds were recruited by contacting the principal of a Waldorf school in Trondheim. Young adults were recruited from Dragvoll University campus at the Norwegian University of Science and Technology (NTNU) and were mostly students.

Young infants had a mean age of 4.5 months (SD = 0.5, range 4–5). Four infants in this group had started to accidentally roll over from back to stomach, while the others had no self-produced locomotor experience. Older infants had a mean age of 10 months (SD = 0.6, range 9–11). At this age, all infants had at least 6 weeks of experience with self-produced locomotion (crawling or creeping). Mean ages of the last three groups were 6 years (SD = 0.4, range 6–7), 12 years (SD = 0.5, range 11–12) and 25 years (SD = 2.5, range 21–29), respectively.

All participants had normal or corrected-to-normal vision and were born full-term (≥ 38 weeks of gestation). An information letter explaining the aim of the study was given to all participants (or parents in case of infants/children) right before signing a consent form. Electroencephalography (EEG) is a non-invasive procedure that has no harmful consequences. Participants were informed that they could withdraw from the study at any point. This study has been approved by the Norwegian Social Science Data Services (NSD) and the Regional Committee for Medical and Health Research Ethics (REC Central). Raw data will be made available on reasonable request.

### Experimental stimuli and paradigm

**Fig. 1:**
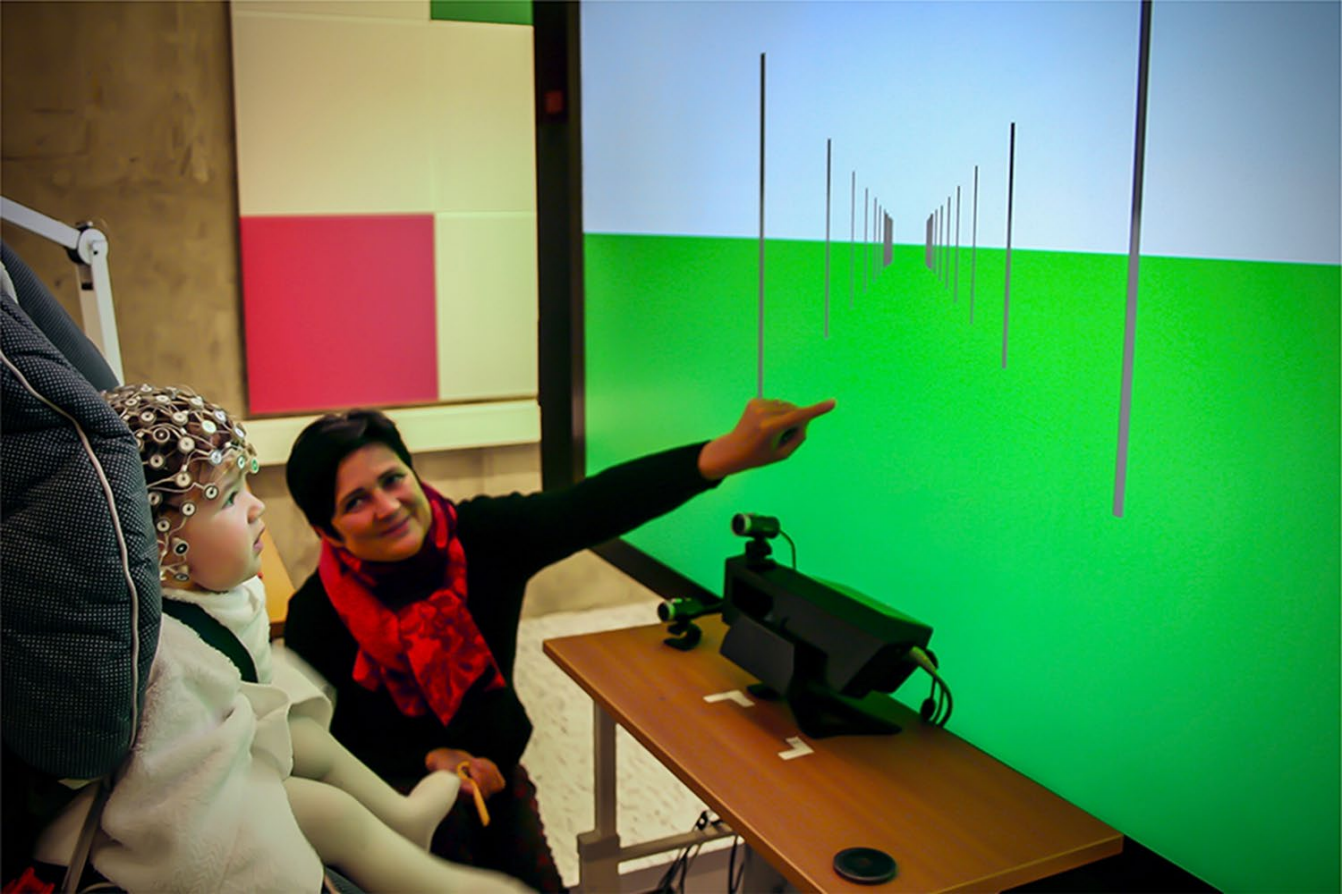
Experimental setup. Experimental room with a 4-month-old infant sitting in a baby car seat in an adjustable chair, wearing an electrode net consisting of 128 sensors. Moving poles simulating forward self-motion through optic flow appeared on the large screen in front of the infant. The eye tracker was placed on the desk, between the participant and the screen, to monitor gaze

E-prime software (Psychological Software Tools, Inc.) generated an optic flow pattern representing a virtual road with moving poles at either side of it, simulating forward motion at three different ecologically valid speeds. The pattern was transmitted onto a Microsoft Surface Hub 84″ (1171.5 mm × 2202.9 mm × 105.4 mm) through an Athen Masterview CS1782 DVI-KVMP switch, with a refresh rate of 60 Hz. The participants were seated at approximately 75 cm distance from the screen so that the screen subtended an angle of 72° by 50°, with a resolution of 1743 pixels per meter (Fig. 1).

The three different motion speeds used were comparable to walking, jogging, and cycling (approximately 5, 10 and 20 km/h), and are referred to as low, medium, and high speed, respectively. When visual motion was presented, several poles started to move outwards from the centre of the screen, simulating forward motion. The simulated pole height was 1.3 m, eye height from the ground was 0.65 m, and simulated distance between poles was 1.69 m. Before the experiment commenced, it was ensured that participants had the same horizon-view facing the centre of the screen. To this end, the chair’s height was adjusted up or down, based on the participant’s stature. Poles that disappeared were immediately replaced at the centre, giving the impression of driving down a road. When simulating higher speed, the number of poles was increased, which effectively decreased the distance between them. A video of the display can be found as supplementary material.

Each motion speed was presented for 1500 ms in a random order. A static control condition of 1500 ms was presented between each motion condition, to prevent motion adaptation. During the static condition, the participants were shown a static frozen picture of the previous motion trial, with the same number of poles. The static trials were also used as control condition in the TSE analysis.

### Data acquisition

An Electrical Geodesic Inc. (EGI) Sensor Net 200 was used to record EEG activity (Tucker 1993). It consisted of an array of 128 Ag/AgCl sponge sensors for infants and 256 sensors for children, adolescents, and adults.

The net was connected to a high-input EGI amplifier set at a maximum impedance of 50 kΩ, to ensure optimal signal-to-noise ratio (Ferree et al. 2001; Picton et al. 2000). A Macintosh computer recorded amplified signals at a sampling rate of 500 Hz (200 Hz low-pass and 0.1 Hz high-pass online filters), using Net Station Acquisition software version 5.4.1.2. To ensure that the participants were looking at the screen, an infrared Tobii X50 eye tracker was used to monitor gaze, and two digital cameras collected behavioural data for offline analysis (Fig. 1). Except for the 12-year-olds and the adults, a parent and experimenter were always sitting next to the participant to provide support in case of need. If participants (mostly infants) were looking away from the screen, the experiment was paused until attention was restored. Recorded data were stored for offline analysis.

### Procedure

The experiment was carried out at the Developmental Neuroscience Laboratory (Nu-Lab) at the Department of Psychology at the Norwegian University of Science and Technology (NTNU) in Trondheim, Norway. All participants arrived some time before the experiment (with their parents, in case of minors) to receive explanations about the procedure. Before the start of the experiment, the babies had the time to get familiar with the environment exploring the laboratory’s playground, and their motor skills were observed to assess their level of experience with self-produced locomotion. None of the younger infants could roll over from back to stomach, while all of the older infants could comfortably creep or crawl and had been doing so for at least six weeks based on parental report. For the 6- and 12-year-olds, parents provided information regarding their children’s general motor competencies. Participants’ heads were measured to select the appropriate net, which was then soaked in a saline solution for at least 10 min to ensure good impedance. Thereafter, the net was slightly towel-dried and placed on the head.

The experiment took place in a dimly lit experimental room, where participants were seated in front of a large screen. Infants were placed in a baby car seat, with a parent always on the side. The net was connected to the amplifier, and the impedance checked in the adjoining control room. In case of poor contact, extra saline solution was added to the net with a plastic pipette. An assistant was present in the room to monitor the experiment and to help infants and children to focus on the screen.

The experiment was carried out in one block, but could be paused when necessary. In case of significant level of distress, boredom, or tiredness, the experiment was ended. The stimuli were presented in a random sequential order for about 115–200 trials and the experiment lasted for 4–7 min. While participating in this study, all participants also took part in other experiments aimed to investigate auditory looming, occlusion, and directions of optic flow. After the session, 6- and 12-year-olds and adults were debriefed and asked for feedback about the experiments. All of them reported to be able to differentiate between the visual motion conditions.

### Brain data analysis

EEG raw data were segmented using Net Station Tools software version 5.4.1.2 and analysed offline with Brain Electrical Source Analysis (BESA, GmbH) version 6.1 research software. Averaging epochs were set from − 200 to 800 ms, with a baseline definition of − 100 to 0 ms.

Physiological artefacts caused by eye blinks or eye movements were semi-automatically removed or re-estimated using spherical spline interpolation (Perrin et al. 1989; Picton et al. 2000). Manual artefact correction was performed with threshold values of 0.1 μV for low signals and 75 μV for gradients, to separate brain activity from eye movement artefacts with spatial filters (Berg and Scherg 1994; Ille et al. 2002). Maximum amplitude was set at 200 μV for all groups except adults, who had a threshold of 120 μV instead. Bad channels were excluded, but not more than 10% in each participant. No more than five electrodes in occipital and parietal areas, or two electrodes adjacent to each other were removed.

If an infant or a child looked away from the screen for more than two trials, the experiment was paused, and the assistant would play with them to revive interest. In that case, the corresponding trials were removed by visual inspection. Since adolescents and adults were able to sit still and focus on the screen for the entire session, no trials were removed from their recordings. Motion trials were more or less equally distributed over the three speeds. No participant had less than 12 accepted trials per single condition.

### VEP peak analysis at the electrode level

Peak analysis at the electrode level was carried out to investigate VEP responses. EEG data of each participant were averaged and interpolated into standard 81-electrode configuration of the 10–10 international system. Spherical line interpolation was used to estimate the signal for the reference free (10–10) montage (Perrin et al. 1989). The individual averages were then combined into a grand average and used as a reference during the selection of the individual N2 components.

Low cut-off filter was set at 1.6 Hz, to remove low drift in the data, while high cut-off filter was set at 80 Hz to remove high-frequency activities. Notch filter was always kept at 50 Hz. All thresholds and filters were set at the same values for every participant. 3D spherical spline whole-head voltage maps were used to select N2 components of the individual averages over occipito-parietal areas by identifying maximum N2 activity for the most dominant waveform. Peak latency was measured from stimulus onset to the peak of each scalp N2 component.

### Time–frequency analysis in brain space

Time–frequency analysis was carried out to investigate changes in amplitude over time. Complex demodulation (Papp and Ktonas 1977) can be used to transform time-domain signals into the time–frequency domain. Data were transformed from electrode level to source montage dipoles by creating a predefined age-appropriate head model template (Hoechstetter et al. 2004; Richards et al. 2015) for each participant, and the artefact-corrected coordinate files were added.

**Fig. 2:**
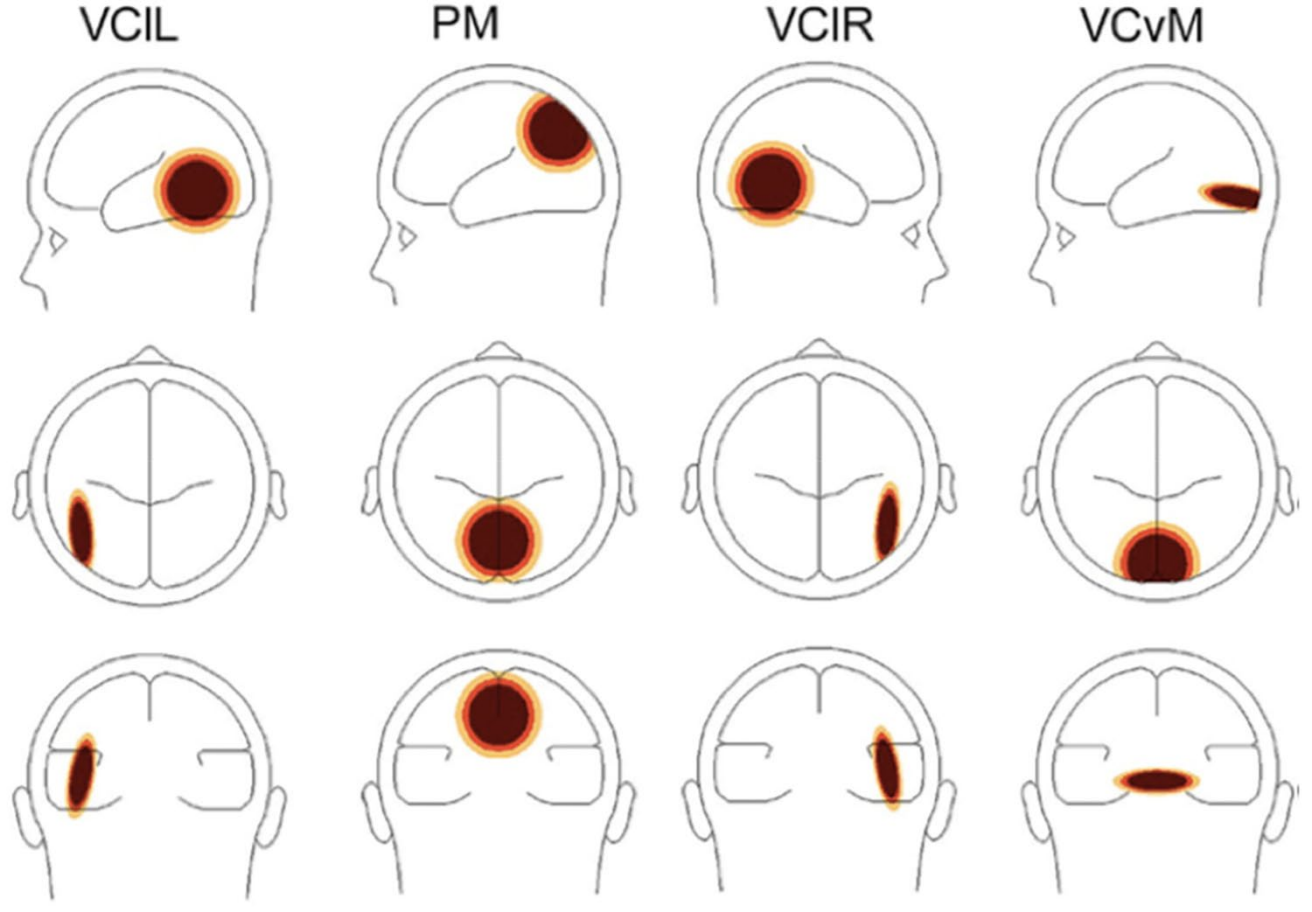
Head model of visual cortical areas of interest with approximate Talairach coordinates (Talairach and Tournoux 1988). From left to right: visual cortex lateral left (VClL), *x* = − 45.2, *y* = − 57.2, *z* = 6.5; parietal midline (PM), *x* = 0.0, *y* = − 72.3, *z* = 37.0; visual cortex lateral right (VClR), *x* = 45.2, *y* = − 57.2, *z* = 6.5; and visual cortex vertical midline (VCvM), *x* = 0.0, *y* = − 84.9, *z* = − 14.3. When a particular brain region is active, the signal magnitude shows the estimated source activity

Measuring oscillatory activity on the scalp surface may be difficult due to the smearing effect of volume conduction in EEG and the nature of dipole fields. Since the resulting scalp waveforms have mixed contributions from underlying brain sources, source montages derived from a multiple source model were used, to obtain optimal separation of focal activity (Scherg and Berg 1991). The analysis involved occipital and parietal areas, as these areas were found to be active during motion stimuli presentation (Probst et al. 1993; Zeki et al. 1991). Visual cortical areas included in the analysis were visual cortex lateral left (VClL), parietal midline (PM), visual cortex lateral right (VClR), and visual cortex vertical midline (VCvM), as shown in Fig. 2.

Bone thickness and bone conductivity were 3 mm and 0.02 σ for infants at 4–5 and 9–11 months, 5 mm and 0.018 σ for 6-year-olds, 6 mm and 0.010 σ for 12-year-olds, and 7 mm and 0.0042 σ for adults (Richards et al. 2015). TSE displays were set from 0 to 40 Hz, with a frequency of 1 Hz and time sample of 50 ms. The epoch was set from − 100 to 1000 ms. Low cut-off filter was set at 1.6 Hz (high-pass), while high cut-off filter was set at 40 Hz (low-pass). All thresholds and filters were the same for every group. Averaged waveforms were removed from the analysis, and power (μV^2^) used as measure unit. Low, medium, and high speeds were first compared to each other and to the static control condition. Similar latencies were found when each of the motion speeds were separately compared with the TSEs of the static control condition. In addition, no significant differences were found when the TSEs were compared between pairs of the three motion speeds, meaning that the oscillatory activity in response to speed was similar, irrespectively of speed. For this reason, the three motion speeds were collapsed into a single motion condition, and compared to the static control condition, and a computation of this comparison was performed. Finally, a bootstrapping method tested significance (*α* = 0.05) in each TSE plot, for each of the participants.

## Results

### VEP responses

The four electrodes with the highest N2 amplitude for low speed were selected in the grand average VEPs and used as a guide to identify the individual N2 component for each participant. The peak latency was then recorded and analysed further. Since the location of the signal may slightly change from subject to subject, we decided to include four electrodes to have a more comprehensive view of the underlying cortical activity. The four chosen electrodes are not representative of single electrodes, but they record the signal from a cluster of electrodes recording the underlying cortical activity. The chosen electrodes were Oz, O2, PO8, and PO10 for infants at 4–5 months; Oz, O2, POz, and PO4 for infants at 9–11 months; Oz, O2, PO8, and POz for 6-year-olds; O2, P4, PO4, and PO8 for 12-year-olds; and Oz, O2, PO4, and POz for adults. Since the electrodes were selected based on which part of the brain responded most strongly to visual motion, their location was slightly different between age groups. However, the selected electrodes were always located in posterior cortical areas for all participants, and they were also adjacent to each other.

**Fig. 3:**
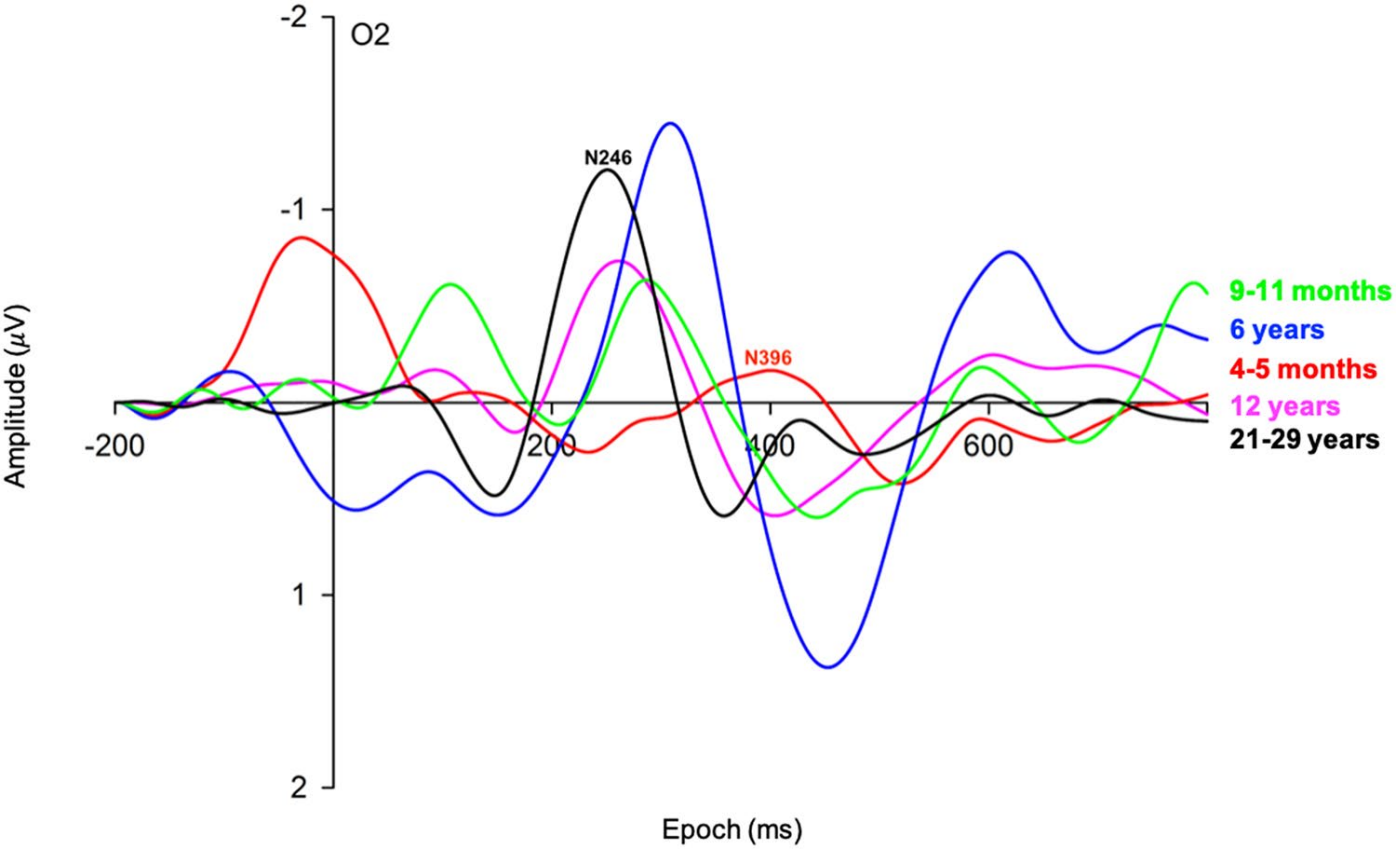
Grand averaged motion VEPs across occipital channel O2 in infants at 4–5 months (red), infants at 9–11 months (green), children at 6 years (blue), adolescents at 12 years (purple), and adults (black). Low, medium and high speeds were collapsed into a single motion condition. Epoch is from − 200 to 800 ms. Actual N2 peak latencies were 396 ms for infants at 4–5 months, 328 ms for infants at 9–11 months, 299 ms for children at 6 years, 278 ms for adolescents at 12 years, and 246 ms for adults, indicating a tendency for latency to decrease with age

Mean motion trial contributions were 57 (SD = 13) for infants at 4–5 months, 56 (SD = 12) for infants at 9–11 months, 105 (SD = 23) for 6-year-olds, 104 (SD = 15) for 12-year-olds, and 103 (SD = 15) for adults (Fig. 3).

**Fig. 4:**
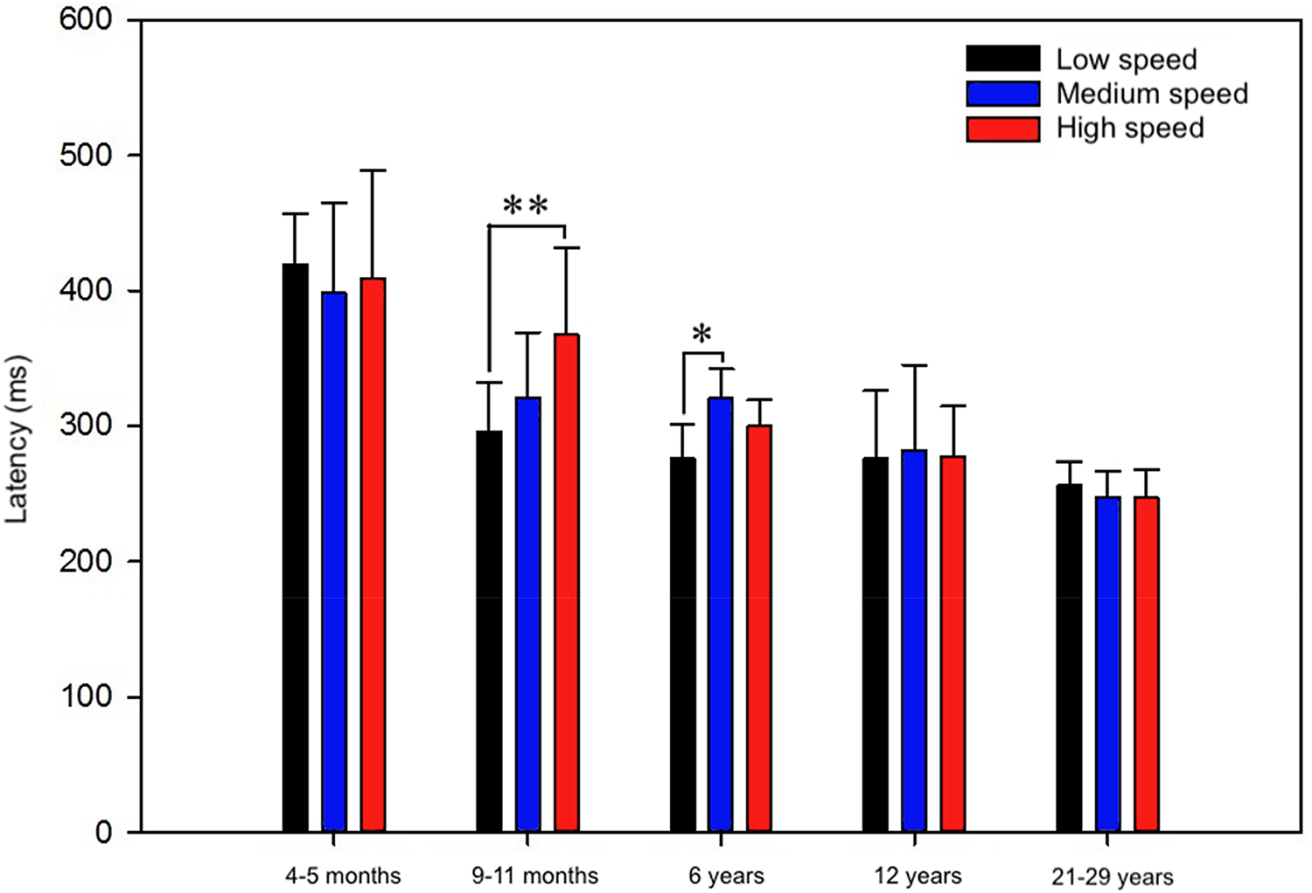
Group means with standard deviation bars of N2 peak latencies for low, medium, and high speeds in infants at 4–5 months and at 9–11 months, 6- and 12-year-old children, and adults. Latency decreased significantly with age. In addition, 9-month-old infants and 6-year-old children showed the shortest latencies for low speed, indicating that they found low speed easier to detect. *Significant at *p* < 0.05; **significant at *p* < 0.01

As shown in Fig. 4, mean peak latencies (ms) for slow, medium, and high speeds were 404 (SD = 52), 398 (SD = 67), and 388 (SD = 73) for infants at 4–5 months and 296 (SD = 36), 321 (SD = 48), and 367 (SD = 65) for infants at 9–11 months, respectively. For 6-year-olds, the corresponding latencies were 276 (SD = 26), 321 (SD = 22), and 300 (SD = 19) ms, while for 12-year-olds they were 276 (SD = 51), 282 (SD = 63), and 277 (SD = 37) ms. For adults, latencies were 246 (SD = 14), 247 (SD = 20), and 247 (SD = 21) ms for low, medium, and high speeds, respectively. For additional data, see Supplementary Figure A with additional graphs of the grand average motion VEPs across two electrodes for each group.

A repeated measure ANOVA was used to test for differences in N2 peak latencies with motion speed (low, medium, high) as within-subject factor and age (4–5 months, 9–11 months, 6 years, 12 years, adults) as between-subject factor. For N2 peak latency, the results showed a significant main effect of age, *F*(4,35) = 31.60, *p* < 0.001, indicating that latency decreased with age.

In addition, post hoc tests for groups using Bonferroni as confidence interval adjustment were carried out. According to the results, infants at 4–5 months showed no differences in latency between motion speeds, while infants at 9–11 months and children at 6 years did: the former between low and high speeds, with significantly shorter latencies for low speed, *F*(2,14) = 4.15, *p* < 0.05, the latter between low and medium speeds, again with shorter latencies for low speed, *F*(2,14) = 10.32, *p* < 0.01. Twelve-year-olds and adults showed similarly short latencies for the three motion speeds. However, overall latencies for adolescents were still higher than those for adults, with N2 mean latency across speeds of 278 ms (SD = 49) for 12-years-olds and 246 ms (SD = 19) for adults, confirming the main effect of age reported above.

### Time–frequency responses

A time–frequency analysis was carried out for the three motion conditions and the static control condition for every participant individually. The visual motion conditions were compared to one another in each of the five groups separately, but no significant differences between speeds were found, indicating a similar induced response to visual motion (see Supplementary Figures B1 to B5 for time–frequency plots and the corresponding probability maps). Hence, motion speeds were collapsed into one motion condition and compared with the static control condition. Significant negative clusters are an indication that motion had significantly lower amplitudes than static.

**Fig. 5:**
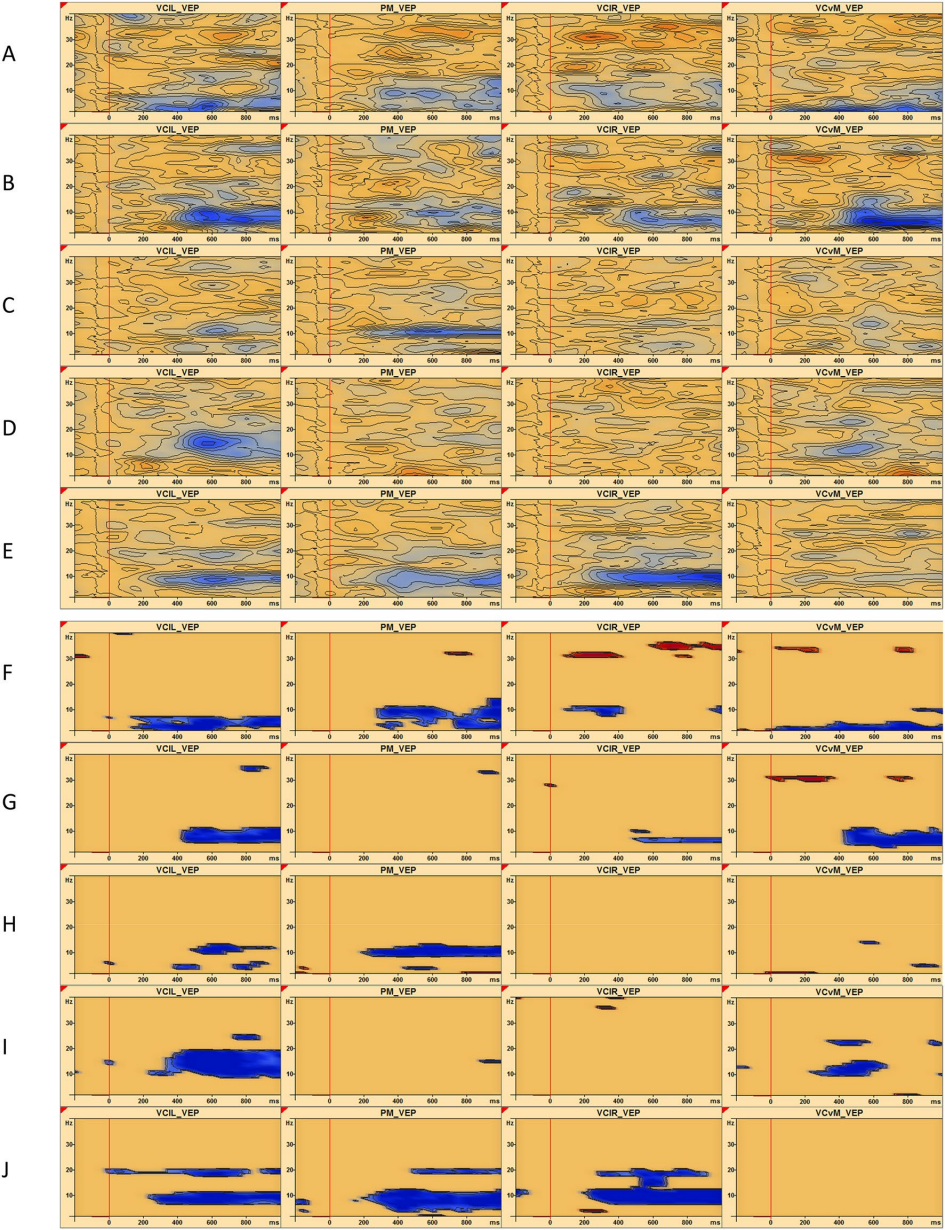
TSE plots (**A**, **B**, **C**, **D**, **E**) and corresponding TSE probability maps (*p* < 0.05; **F**, **G**, **H**, **I**, **J**) in sources: visual cortex lateral left (VClL), parietal midline (PM), visual cortex lateral right (VClR), and visual cortex vertical midline (VCvM), when visual motion was compared to static non-flow in five typical participants, one from each age group. From top to bottom, infant at 5 months (**A**, **F**), infant at 10 months (**B**, **G**), 6-year-old child (**C**, **H**), 12-year-old adolescent (**D**, **I**), and 26-year-old adult (**E**, **J**). Frequency (0–40 Hz) is represented on the *y*-axis, while latency (− 200 to 1000 ms) is on the *x*-axis. The vertical red line marks stimulus onset at 0 ms. Epoch is from − 200 to 800 ms, with a baseline from − 100 to 0 ms. In the TSE plots (**A**, **B**, **C**, **D**, **E**), blue areas indicate induced desynchronized activity (with decreased spectral amplitude), while red areas show induced synchronized activity (with increased spectral amplitude). Probability maps (**F**, **G**, **H**, **I**, **J**) show the areas with the largest change in activity. Compared to baseline, infants at 4–5 months showed significant theta and alpha activity (**F**); infants at 9–11 months showed significant alpha activity (**G**); 6- and 12-year-olds showed significant activity in the alpha and beta range (**H**, **I**). Finally, adults showed significant beta activity compared to baseline (**J**)

Figure 5F–J shows probability maps for visual motion compared to the static condition for five typical participants, one from each age group. Significant differences (*p* < 0.05) were found between motion compared to baseline in the theta and alpha bands for infants at 4–5 months (Fig. 5F), in the alpha band for infants at 9–11 months (Fig. 5G), and in the alpha and beta bands for 6- and 12-year-old children (Fig. 5H, I). Finally, adults showed a decrease in amplitude in the beta band compared to the baseline (Fig. 5J).

In response to visual motion, infants at 4–5 months showed desynchronized oscillatory activity in the theta (4–8 Hz) and alpha (8–12 Hz) bands (Fig. 5A). Infants at 9–11 months showed desynchronized oscillatory alpha (8–12 Hz) activity (Fig. 5B). Six- and 12-year-olds showed desynchronized oscillatory activity in the alpha (8–12 Hz) and beta (12–30 Hz) bands (Fig. 5C, D). Finally, adults showed desynchronized oscillatory beta (12–30 Hz) activity (Fig. 5E). In contrast, induced synchronization was found in response to the static condition in the alpha- and beta-bands in all participants. Activity was usually observed around 200–800 ms.

## Discussion

In the present cross-sectional study, high-density EEG was used to study brain electrical activity in response to simulated forward visual motion in infants at 4–5, infants at 9–11 months, children at 6 years, adolescents at 12 years, and young adults, to investigate the development of speed perception from infancy to early adulthood. Analyses on evoked and induced responses to visual motion were performed on EEG data to investigate how visual motion perception develops across the lifespan.

### Evoked responses to speed of motion

Infants at 4–5 months displayed significantly longer N2 latencies overall of around 400 ms. Moreover, it was found that they showed equally long N2 responses for the three motion speeds, indicating that they were sensitive to visual motion as such, but could not differentiate between motion speeds. The slower information transmission may be caused by the absence of a fully myelinated system (Braddick et al. 2003), which leads to longer latencies as result of a less specialized neuronal network (Agyei et al. 2015; Dubois et al. 2006; Howard et al. 1996; Johnson 2000).

Infants at 9–11 months displayed shorter latencies when compared to prelocomotor infants, and they could differentiate between visual motion speeds. Similar results were found in another study (Vilhelmsen et al. 2019), where it was reported that infants at 4–5 months could not differentiate between visual motion speeds and directions, whereas crawling 8- to 11-month-old infants could. The perception of speed develops during the first year of life due to the infant’s motor experience (James and Swain 2011), with higher speeds resulting in higher latencies (Kawakami et al. 2002). The present study corroborates these findings, where crawling infants at 9–11 months showed significantly longer latencies for high speed compared to low speed.

Improved visual motion perception in infants as reported in the present study could be the result of several developmental factors, such as the rapid growth and overproduction of cortical synapse formation in most cortical areas during the first 2 years of life (Casey et al. 2000) and the maturation of local glucose metabolic rates in posterior temporal and parietal lobes, that raise the effectiveness of motion information transmission and processing (Chugani et al. 1996). In addition, the increasing neuronal myelination of connecting fibres (Grieve et al. 2003) and the increasing maturity of visual pathways during the first year of life (Agyei et al. 2016b; Van der Meer et al. 2008) could lead to the relatively faster processing of visual motion and to the shorter latencies found in older infants.

However, brain maturation is not likely the only factor in the development of visual motion perception since a close link between self-generated actions and optic flow processing was found (Agyei et al. 2016a, b; James and Swain 2011). Previous research showed that the passive form of visual flow that prelocomotor infants experience when carried around by a caregiver or in a stroller leads to only a minimum level of improvement in their sensitivity to optic flow (Gilmore et al. 2004; Raudies et al. 2012), while when infants start to crawl, they become able to explore the surroundings, relying more on structured information for an effective navigation (Bruggeman and Warren 2010; Bruggeman et al. 2007; Lee and Aronson 1974; Slobounov et al. 2006). Therefore, it is reasonable to assume that, in addition to brain maturation, the active experience of self-produced locomotion could have led to the shorter latencies found in older and mobile infants.

Compared to infants, 6-year-old primary school children showed significantly shorter latencies for visual motion, and could differentiate between low and medium speed. Around this age, children experience more advanced forms of locomotion, such as running and cycling. The observed decrease in latency could indicate an improvement in perception–action responses (Gilmore et al. 2004; James and Swain 2011). However, a study on fear and danger appraisals of a road-crossing scenario (Rosenbloom et al. 2008), reported that pre-schoolers could not properly realize the danger associated with speed, and they experienced also difficulties evaluating direction and proximity of moving vehicles, probably due to their short stature. Moreover, they may not be able to detect vehicles approaching at speeds in excess of 32 km/h (Wann et al. 2011). In the present study, young children’s latencies were still higher compared to the 12-year-olds, suggesting a visual system not yet fully developed.

Adolescents and adults displayed similar N2 latencies for low, medium, and high speeds of around 275 and 250 ms across motion speeds, respectively. The current results may indicate that since 12-year-olds and adults have more experience with these speeds, being exposed to them daily, they may have perceived them as equally easy to detect. Yet, 12-year-olds displayed longer latencies than adults, who showed significantly shorter latencies overall. Since an adult-level response to visual motion does not occur until 16–18 years of age (Hadad et al. 2015; Joshi and Falkenberg 2015), the present findings may suggest that myelination is still going on in the adolescent brain.

### Induced responses to visual motion

TSEs showed differences between the five age groups when comparing visual motion to static non-flow. Desynchronized activity in all the visual sources was found in response to visual motion in every participant, indicating that underlying neuronal cell assemblies fired in desynchrony, while induced synchronization in the alpha- and beta-bands was found in response to static non-flow, with cells firing in synchrony. Similar desynchronized activity in response to visual motion was found in previous studies (Agyei et al. 2016b; Van der Meer et al. 2008; Vilhelmsen et al. 2019), and is considered to reflect a preparation for information processing (Pfurtscheller 1992), while synchronized activity in response to visual motion reflects a resting state or not-active neuronal assemblies (Pfurtscheller et al. 1996).

According to previous research, infants respond to visual motion with low-frequency activity (Agyei et al. 2016b; Stroganova et al. 1999), due to the immaturity of their visual system (Carmeli et al. 2005; Orekhova et al. 2006; Van der Meer et al. 2008). In the present study, infants at 4–5 months showed oscillatory activity in the theta- and alpha-bands in response to visual motion. Theta and alpha desynchronizations are associated with increased task complexity and more efficient task performance, which require more attention (Pfurtscheller and Lopes da Silva 1999). Infants at 9–11 months showed oscillatory activity only in the alpha-band. The progression from lower to higher frequencies in response to visual motion has been linked to an increase in visual experience in infants from 7 to 11 months of age (Orekhova et al. 2006; Stroganova et al. 1999). A previous study used the same stimulus with higher speeds and included forwards and backwards simulated self-motion, and found that infants at both 4–5 and 8–11 months displayed theta- and alpha-band oscillations in response to visual motion (Vilhelmsen et al. 2019). The present study corroborates these findings, where infants during the first year of life showed low-frequency oscillatory brain activity in response to visual motion, with an increase from theta- to alpha-band activity as they got older.

Six- and 12-year-olds showed oscillatory desynchronized activity in the alpha- and beta-bands, indicating a progression from the larger and less specialized oscillatory cell assemblies of infants to fewer and more specialized neuronal networks with increasing age, in accordance with previous studies (Van der Meer et al. 2008; Vilhelmsen et al. 2015).

Finally, adults showed beta-band desynchronization when visual motion was compared to static non-flow, indicating a fully developed visual perception system (Babiloni et al. 2002; Neuper et al. 2006; Pfurtscheller et al. 1994; Van der Meer et al. 2008).

## Conclusions

This study has shown the development of speed perception from infancy to early adulthood for ecologically valid motion speeds corresponding to walking, jogging, and cycling. N2 latencies decreased significantly with age, from around 400 ms for young infants at 4–5 months to 250 ms for adults. Prelocomotor infants were not able to differentiate between visual motion speeds, while older infants with locomotor experience and children at 6 years did, with shortest latency for low speed. 12-year-olds and adults showed similar short latencies for the three motion speeds, indicating that they perceived them as equally easy to detect. However, latencies for adolescents were still slightly but significantly higher than those for young adults, suggesting that myelination is still going on in the adolescent brain. Time–frequency analysis indicated that with increasing age, participants showed a progression from low- to high-frequency desynchronized oscillatory activity in response to visual motion. We conclude that self-produced locomotor experience is a prerequisite for perceiving speed of motion and that children’s motion speed perception is not fully developed until they reach adolescence. Therefore, educational interventions among young pedestrians should be considered to maximize children’s road safety and prevent road accidents.

## Supplementary Information

Below is the link to the electronic supplementary material.

Supplementary file1 (MOV 80311 KB)

Supplementary Figure A: Grand average motion VEPs across occipital (O2) and parietal (PO4 or PO8) electrodes in infants at 4–5 months (A), infants at 9–11 months (B), 6-year-olds (C), 12-year-olds (D), and adults (E). Latencies are displayed on the x-axis, while amplitudes are on the y-axis. Epoch is from -200 to 800 ms. Actual N2 mean peak latencies (in ms) for low (black), medium (blue), and fast (red) speed were 419, 398, and 409 for infants at 4–5 months, and 296, 321, and 367 ms for infants at 9–11 months, respectively. For 6-year-olds, the corresponding latencies were 276, 321, and 300 ms, while for 12-year-olds they were 276, 282, and 277 ms. For adults, latencies were 256, 247, and 247 ms for low, medium, and high speed, respectively. N2 peak latencies decreased significantly with age, from around 400 ms for young infants at 4–5 months to 250 ms for adults (DOCX 5541 KB)

Supplementary Figure B1: TSE plots (A, B, C) and corresponding TSE probability maps (D, E, F; p < 0.05) in sources VClL, PM, VClR, and VCvM when visual motion was compared to static non-flow in a typical participant aged 5 months. From top to bottom, low speed (A), medium speed (B), and high speed (C), compared to static non-flow. The vertical red line marks stimulus onset at 0 ms. Epoch is from -200 to 800 ms, with a baseline from -100 to 0 ms. In the TSE plots (A, B, C), blue areas indicate induced desynchronized activity with decreased spectral amplitude, while red areas show induced synchronized activity with increased spectral amplitude. Probability maps (D, E, F) show the areas with the largest change in activity. Compared to baseline, the participant aged 5 months showed significant theta and alpha activity (DOCX 2937 KB)

Supplementary Figure B2: TSE plots (A, B, C) and corresponding TSE probability maps (D, E, F; p < 0.05) in sources VClL, PM, VClR, and VCvM when visual motion was compared to static non-flow in a typical participant aged 10 months. From top to bottom, low speed (A), medium speed (B), and high speed (C), compared to static non-flow. The vertical red line marks stimulus onset at 0 ms. Epoch is from -200 to 800 ms, with a baseline from -100 to 0 ms. In the TSE plots (A, B, C), blue areas indicate induced desynchronized activity with decreased spectral amplitude, while red areas show induced synchronized activity with increased spectral amplitude. Probability maps (D, E, F) show the areas with the largest change in activity. Compared to baseline, the participant aged 10 months showed significant alpha activity (DOCX 2839 KB)

Supplementary Figure B3: TSE plots (A, B, C) and corresponding TSE probability maps (D, E, F; p < 0.05) in sources VClL, PM, VClR, and VCvM when visual motion was compared to static non-flow in a typical participant aged 6 years. From top to bottom, low speed (A), medium speed (B), and high speed (C), compared to static non-flow. The vertical red line marks stimulus onset at 0 ms. Epoch is from -200 to 800 ms, with a baseline from -100 to 0 ms. In the TSE plots (A, B, C), blue areas indicate induced desynchronized activity with decreased spectral amplitude, while red areas show induced synchronized activity with increased spectral amplitude. Probability maps (D, E, F) show the areas with the largest change in activity. Compared to baseline, the participant aged 6 years showed significant alpha and beta activity (DOCX 2448 KB)

Supplementary Figure B4: TSE plots (A, B, C) and corresponding TSE probability maps (D, E, F; p < 0.05) in sources VClL, PM, VClR, and VCvM when visual motion was compared to static non-flow in a typical adolescent aged 12 years. From top to bottom, low speed (A), medium speed (B), and high speed (C), compared to static non-flow. The vertical red line marks stimulus onset at 0 ms. Epoch is from -200 to 800 ms, with a baseline from -100 to 0 ms. In the TSE plots (A, B, C), blue areas indicate induced desynchronized activity with decreased spectral amplitude, while red areas show induced synchronized activity with increased spectral amplitude. Probability maps (D, E, F) show the areas with the largest change in activity. Compared to baseline, the participant aged 12 years showed significant alpha and beta activity (DOCX 2532 KB)

Supplementary Figure B5: TSE plots (A, B, C) and corresponding TSE probability maps (D, E, F; p < 0.05) in sources VClL, PM, VClR, and VCvM when visual motion was compared to static non-flow in a typical adult participant aged 26 years. From top to bottom, low speed (A), medium speed (B), and high speed (C), compared to static non-flow. The vertical red line marks stimulus onset at 0 ms. Epoch is from -200 to 800 ms, with a baseline from -100 to 0 ms. In the TSE plots (A, B, C), blue areas indicate induced desynchronized activity with decreased spectral amplitude, while red areas show induced synchronized activity with increased spectral amplitude. Probability maps (D, E, F) show the areas with the largest change in activity. Compared to baseline, the adult participant showed significant beta activity (DOCX 2434 KB)
