# Supplementary material for: Development of motion speed perception from infancy to early adulthood: a high-density EEG study of simulated forward motion through optic flow
Source: Exp Brain Res. 2021 Aug 21;239(10):3143–54. doi: 10.1007/s00221-021-06195-5 (PMC8536648; doi:10.1007/s00221-021-06195-5)
Supplement: Supplementary file 4 [file 221_2021_6195_MOESM5_ESM.docx]

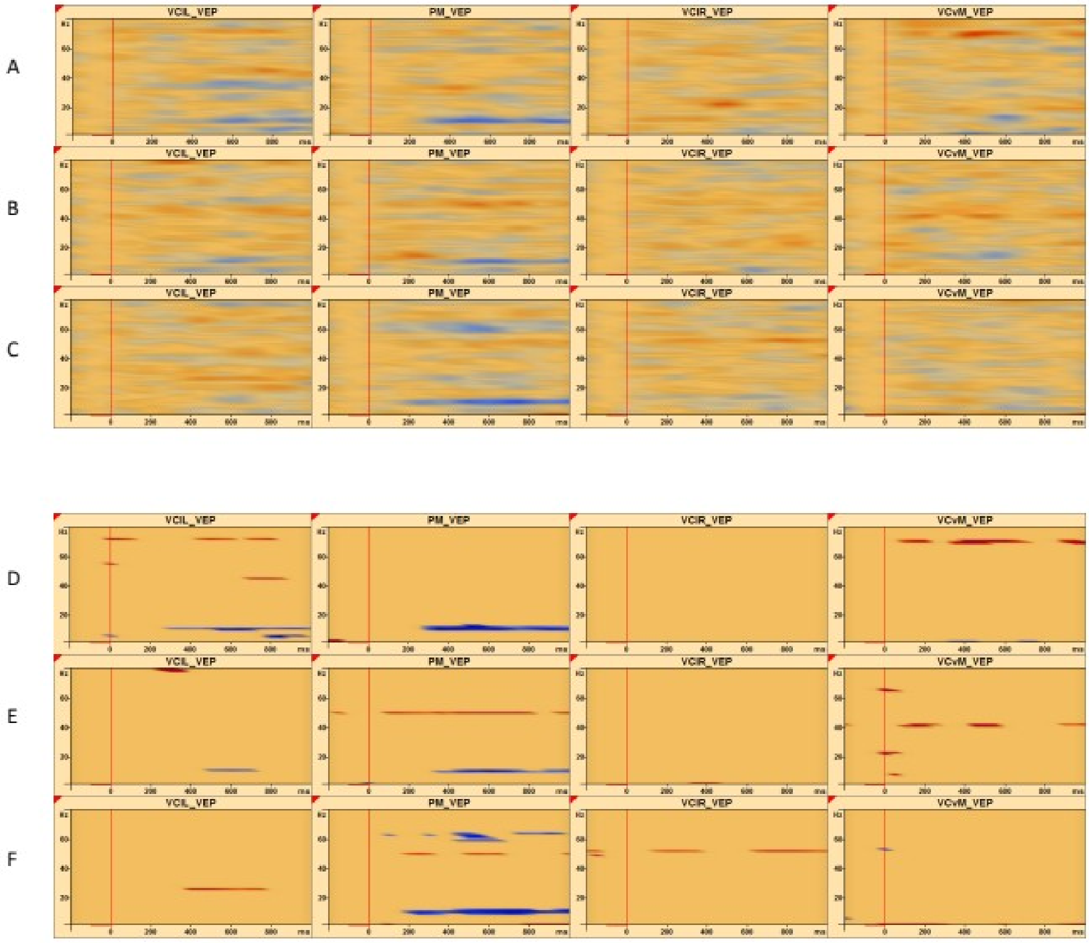
A
B
C
D
E
F
VCIL_VEP
PM_VEP
VCIR_VEP
VCvM_VEP
Hz
ms
